# The prevalence and correlates of oral sex among low-tier female sex workers in Zhejiang province, China

**DOI:** 10.1371/journal.pone.0238822

**Published:** 2020-09-08

**Authors:** Xin Zhou, Qiaoqin Ma, Xiaohong Pan, Lin Chen, Hui Wang, Tingting Jiang

**Affiliations:** Department of HIV/STD Control and Prevention, Center for Disease Control and Prevention of Zhejiang Province, Hangzhou, PR China; University of Westminster, UNITED KINGDOM

## Abstract

**Objectives:**

Oral sex and its associated factors among low-tier female sex workers (FSWs) have not been documented in the Chinese literature. Here, we report this perspective in this group.

**Methods:**

The data were derived from a large cross-sectional study conducted among low-tier FSWs using a structured questionnaire in 21 counties in Zhejiang province, China. The prevalence of oral sex and its associated factors among 2645 low-tier FSWs were analyzed using bivariate and multivariate analysis.

**Results:**

Of all participants, 579 (21.9%) had performed oral sex with clients over the previous month. Multivariate analysis revealed that oral sex is related to being unmarried, low income, early initiation of commercial sex, having conducted commercial sex in more counties, longer duration of commercial sex, larger number of clients, ever having engaged in anal sex, less use of condoms and oral contraceptives during the previous month, low rate of adoption for contraception at the present time, and STI-related symptoms during the previous half year.

**Conclusion:**

Oral sex practitioners among low-tier FSWs in China are at a higher risk of STI, HIV, and unwanted pregnancy compared to those who did not engage in oral sex. Behavioral interventions carried out among low-tier FSWs should specifically target low-tier FSWs who practice oral sex, should carefully take into account the characteristics of these FSWs, provide risk awareness education and training for condom use negotiation, and promote the availability of condom and reproductive health care, timely diagnosis, and treatment of STIs.

## Introduction

Female sexual workers (FSWs) are a high-risk population for human immunodeficiency (HIV) infections, and require intervention compared to the general female population. The risk of HIV infection among FSWs is 13.5 times higher than that of the general female population in low- and middle-income countries [1], and the prevalence of STIs is high among FSWs [2–4]. In terms of working place, FSWs can be categorized into low-, middle-, and high-tier levels based on their different socio-economic levels, types of client, price charged, etc., with low-tier FSWs lying at the lower socio-economic level, working in lower sex trade places such as roadside shops and on-street small venues, and usually providing sexual services to clients at a similar socio-economic level, such as those who are aged, poorer, and less educated, and for a low price sexual trade fee [5–7].

To earn more money, low-tier FSWs must conduct sex with more clients, and provide high-risk sexual services [6, 7]. Moreover, condom use negotiation with clients is difficult for this group, resulting in less condom use among low-tier FSWs [8, 9]. Lower self-protection awareness, less condom use, low access to clinics, and high mobility of sex trade are all problems faced by low-tier FSWs related to HIV/STI transmission [5, 6, 10, 11]. According to China national HIV/AIDs sentinel surveillance report from 2011 to 2015, low-tier FSWs exhibited higher than middle- and high-tier FSWs in HIV and syphilis prevalence, with a HIV prevalence of 0.1–0.2% in middle- and high-tier FSWs versus 0.4–0.6% in low-tier FSWs, and a syphilis prevalence of 1.6–1.9% in middle- and high-tier FSWs versus 3.6–4.6% in low-tier FSWs. A high prevalence of HIV and syphilis was found among Chinese low-tier FSWs [12, 13]. Therefore, low-tier FSWs play an important role in facilitating HIV and STI transmission, and their high mobility could also introduce HIV/STI transmission from one place to another.

Oral sex is one of the most common sexual activities in commercial sexual services provided by low-tier FSWs, with studies on older male clients of FSWs in the US [14] and Peru [15] reporting that over 95% had engaged in oral sex with FSWs or clients over their lifetime. One Chinese study documented that 43.5% of FSWs had practiced oral sex [16], with the prevalence of Chinese heterosexual STD clinic attendees at 6.9% [17]. Some studies have also reported the prevalence of oral sex among common Chinese people. For example, a study in Guangzhou reported that 63.7% of women aged 20–50 had practiced oral sex in their lifetime [18]. The prevalence of oral sex is increasing among Chinese people, with the rate for fellatio at 16.2%, 30.4%, 35.2%, and 36.8%, respectively, in 2000, 2006, 2010, and 2015, while it was 20.3%, 32.6%, 38.8%, and 36.8%, for penilingus [19]. The rate of oral sex ever practiced by sexually active college students [20] and male college graduates [21] is 35.5% and 20.2%, respectively.

Although oral sex has been considered lower HIV-risk sexual behavior compared to vaginal and anal sexual behavior, evidence has shown that unprotected oral sex could be a route for HIV infection [22, 23], as well as STI infection, such as HPV, HSV, gonorrhea, syphilis, Chlamydia trachomatis, chancroid, and Neisseria meningitidis [24–27]. Chinese studies have exhibited that oral sex is an important marker for a profile of high-risk sexual behavior. Those who practiced oral sex (versus those who did not) among heterosexual STD clinic attendees were more likely to initiate sex early, to have multiple sexual partners, and to conduct more frequent sex [17]. Oral sex practitioners among male college graduates have more sexual partners, more casual sexual partners, more sexual partners from other colleges, and from society, and use condoms less than those who do not practice oral sex [21]. FSWs, particularly low-tier FSWs, are at high risk of HIV/STI infection due to their frequent exposure to different sexual clients. However, there are few data on the characteristics of oral sex practitioners and their role in HIV/STI transmission among lower-tier FSWs in China. Therefore, it is important to investigate oral sex and related factors among low-tier FSWs, to help understand and create targeted intervention for this population.

## Materials and methods

### Location and participants

The participants in this study came from a large cross-sectional study conducted in 21 counties that implemented the AIDS Care project in Zhejiang Province, from September to November 2013. The research method and a brief introduction to Zhejiang province and the AIDS Care project have already been introduced elsewhere [6].

FSWs who met the recruitment criteria were regarded as lower-tier FSWs, who were recruited as research participants if they were currently engaging in sex work on the street and/or at small venues, including hair salons, roadside shops, and other venues with fewer than nine FSWs. All the counties participating in this study conducted a survey to confirm the location of low-tier FSWs in their area and then developed a plan to complete this study.

In total, 2648 low-tier FSWs participated. Of these, 2645 FSWs who responded to the question “Have you engaged in oral sex with clients during the past month?” were included in the study analysis.

All participants who met the recruitment criteria were invited to voluntarily participate in the study, and informed of the study’s purpose, method, and that their privacy and confidentiality would be strictly protected. The interviews were conducted in a private, quiet space within the venues. The staff of local centers for disease control and prevention (CDCs), who were trained by research teams prior to the study, interviewed the participants using an anonymous questionnaire. Considering the low education background of the participants, oral consent was obtained from each participant, then recorded by ticking the box following the Chinese word “agree” at the beginning of the questionnaire. The study protocol including consent procedure was approved by the ethics committee of Zhejiang provincial center for disease prevention and control.

### Questionnaire development and measures

The questionnaire used in this study was developed based on instruments used for HIV sentinel surveillance among FSWs and reviews of domestic and foreign literature regarding low-tier FSWs; it was then revised once more through repeated discussions within the research team and consultation with the staff of the CDCs who were responsible for behavioral interventions with FSWs in the counties studied. Finally, the questionnaire was modified through two pilot surveys with low-tier FSWs in two counties.

Self-reported oral sex with commercial clients during the previous one month was used as a dependent variable in the analysis. The independent variables included sociodemographic characteristics, factors related to sexual behavior, HIV/STI risk perception, and self-efficacy scale regarding condom use. The scale measuring self-efficacy regarding condom use consisted of three statements addressing whether a participant could persuade a client to use a condom when a client refused to do so during a sexual encounter, whether she could refuse sex when a client refused to use a condom, and whether she could insist on using a condom with clients every time. The possible responses were “I can,” “I can’t,” and “I’m not sure.” The scores for this scale ranged from 0 to 3, with 3 reflecting a high level of self-efficacy, 1–2 reflecting a middle level of self-efficacy, and 0 reflecting a low level of self-efficacy. The Cronbach’s alpha coefficient for this scale was 0.923.

### Statistical analysis

Data were analyzed using SPSS for Windows (Version 17.0; SPSS Inc., Chicago, IL, USA). The prevalence of self-reported oral sex and the frequency distributions of the independent variables were determined using bivariate analysis. The association between the dependent variable and each independent variable was computed using an odds ratio (OR) with a corresponding 95% confidence interval (95% CI) and a *P*-value based on a chi-square test of proportions. Variables identified as significantly associated with self-reported oral sex in the bivariate analyses were then entered into the logistic regression model to determine the independent contribution of each factor to predicting self-reported oral sex. A backward elimination procedure was used with a *P* value of >0.10 as the removal criteria. Chi-square analysis was used to compare the difference between those who performed oral sex and those who did not. A *P*-value of less than 0.05 was considered statistically significant for these bivariate and multivariate analyses.

## Results

### Sociodemographic characteristics

The sociodemographic characteristics of the oral-sex group and non-oral sex group are described in Table 1. In total, of the 2645 participants, 31.8% were 25 years or younger, while 27.4% were aged over 35 years. Overall, 10.2% were from the local area. Regarding education, 36.8% had received, at most, primary school education, while 53.1% had received junior high school education; 28.7% were unmarried and 62.5% were married or cohabiting. In terms of financial status, 28.2% earned an income of less than 3000 Yuan per month, while 40.4% earned 3000–4000 Yuan (one Yuan is equal to approximately 0.14 US dollars). The working venues for respondents were the street (15.6%), hair salons (63.5%), roadside shops (10.5%), and other (10.2%).

**Table 1:** Demographic characteristics of low-tier female sex worker (n = 2645).

Variable	Oral sex (N,%)*	Non-oral sex (N,%)*	Total (N,%)*
Age			
≤25	232(40.1)	610(29.5)	842(31.8)
26–35	233(40.2)	837(40.5)	1070(40.5)
≥36	114(19.7)	612(29.6)	726(27.4)
Residence			
Local area	68(11.7)	202(9.8)	270(10.2)
Not local area	41(88.3)	1864(90.2)	2375(89.8)
Education			
Primary school and below	185(32.0)	788(38.1)	973(36.8)
Junior high school	341(58.9)	1064(51.5)	1405(53.1)
High school and above	52(9.0)	210(10.2)	262(9.9)
Marital status			
Unmarried	230(39.7)	530(25.7)	760(28.7)
Married/had cohabited	313(54.1)	1340(64.9)	1653(62.5)
Widowed/divorced	36(6.2)	192(9.3)	228(8.6)
Income per month			
<3000	206(35.6)	540(26.1)	746(28.2)
3000–4000	205(35.4)	863(41.8)	1068(40.4)
>4000	161(27.8)	586(28.4)	747(28.2)
Location of sampling			
Street	59(10.2)	353(17.1)	412(15.6)
Hair salon	356(61.5)	1324(64.1)	1680(63.5)
Roadside	93(16.1)	184(8.9)	277(10.5)
Other	71(12.3)	200(9.7)	271(10.2)

*The percentage may not add up to 100% due to missing data.

In total, 579 participants (21.9%) had performed oral sex with clients over the previous month, while 2666 (78.1%) had not. Of those who had performed oral sex in this time period, 81.0% (469) had also conducted vaginal sex, 16.4% (329) had also performed both vaginal sex and anal sex, 0.3% (2) had also performed anal sex with a client, and 2.2% (13) had only performed oral sex with a client (Table 2).

**Table 2:** Categories of oral sex among low-tier female sex worker (n = 579).

Types of sex	N	%
Oral+vaginal	469	81.0
Oral+vaginal+anal	95	16.4
Oral+anal	2	0.3
only oral	13	2.2

Bivariate analysis indicated that participants’ residence was not associated with self-reported oral sex. The FSWs aged 26–35 years (OR = 0.73, 95% CI = 0.59–0.90) and over or equal to 36 years (OR = 0.49, 95% CI = 0.38–0.63) compared with those under 26 years, those who were married or cohabiting (OR = 0.54,95%CI = 0.44–0.66), and those who were widowed or divorced (OR = 0.43, 95% CI = 0.29–0.64) were negatively associated with oral sex compared with those who were unmarried, and those who earned 3000–4000 RMB (OR = 0.62, 95% CI = 0.50–0.78), and over 4000 RMB (OR = 0.72, 95% CI = 0.57–0.91). The FSWs who had received junior high school education (OR = 1.37, 95% CI = .12–1.67) were positively associated with oral sex compared with those who had received primary school education at most, those who worked from hair salons (OR = 1.61, 95% CI = 1.19–2.17), roadside shops (OR = 3.02, 95% CI = 2.09–4.39), or other (OR = 2.12, 95% CI = 1.44–3.13), or those who worked on the streets.

### Correlates of self-reported oral sex during the previous month

Bivariate analysis indicated that the FSWs who were negatively associated with oral sex were those who had initiated commercial sex at the age of 26–30 years (OR = 0.58, 95% CI = 0.44–0.75) or over 30 years (OR = 0.28, 95% CI = 0.20–0.38) versus those who had initiated sex at the age of 20 or less than 20; those who always used condoms versus those who never/sometimes used condoms (OR = 0.49, 95% CI = 0.41–0.59); those who always used oral contraceptives versus those who never/sometimes used them (OR = 0.27, 95% CI = 0.16–0.43); those who used contraception versus those who did not use them (OR = 0.81, 95% CI = 0.67–0.97)(Contraception refers to intrauterine devices, tubal ligation, or the Norplant method; those FSWs who adopted one of these measures were considered to be using contraception); and those who had a score of 1–2 (OR = 0.68, 95% CI = 0.51–0.92) or 3 (OR = 0.58, 95% CI = 0.48–0.71) on the condom-use self-efficacy scale versus those who had a score of 0 on this scale were negatively associated with experience of oral sex (Table 3).

**Table 3:** Correlates of oral sex during the previous month among low-tier female sex worker (n = 2645).

Variable	Total (N, %)	Oral sex (%)		Crude OR (95%CI) *	P value
Age of first commercial sex					
<21	551(20.8)	164(29.8)		1	
21–25	886(33.5)	232(26.2)		0.84(0.66–1.06)	0.140
26–30	628(23.7)	123(19.6)		0.58(0.44–0.75)	0.000
>30	563(21.3)	59(10.5)		0.28(0.20–0.38)	0.000
County number for commercial sex					
1	1165(44.0)	141(12.1)		1	
2	630(23.8)	140(22.2)		2.08(1.60–2.68)	0.000
>2	847(32.0)	298(35.2)		3.94(3.15–4.94)	0.000
Duration of commercial sex					
1-12months	1067(40.3)	131(12.3)		1	
13–24 months	428(16.2)	133(31.1)		3.22(2.45–4.24)	0.000
>24 months	1146(43.3)	315(27.5)		2.71(2.17–3.39)	0.000
Number of commercial clients during the previous one month					
<16	972(36.7)	104(10.7)		1	
16–30	855(32.3)	280(32.7)		4.06(3.17–5.21)	0.000
>30	799(30.2)	194(24.3)		2.68(2.06–3.47)	0.000
Anal sex during the previous one month					
No	2512(95.0)	482(19.2)		1	
Yes	133(5.0)	97(72.9)		11.35(7.64–16.85)	0.000
Condom use during the previous one month					
Never/sometimes	1290(48.8)	362(28.1)		1	
Always	1354(51.2)	217(16.0)		0.49(0.41–0.59)	0.000
Oral contraceptive use during the previous one month					
Never/sometimes	2400(90.7)		560(23.3)	1	
Always	241(9.1)		18(7.5)	0.27(0.16–0.43)	0.000
Contraception measures at present time					
No	1305(49.3)		310(23.8)	1	
Yes	1337(50.5)		269(20.1)	0.81(0.67–0.97)	0.024
STI related symptom during the previous half year					
No	2319(87.7)		467(20.1)	1	
Yes	317(12.0)		110(34.7)	2.11(1.64–2.71)	0.000
Having seen a doctor during the previous half year					
No	1876(70.9)		394(21.0)	1	
Yes	768(29.0)		185(24.1)	1.19(0.98–1.46)	0.082
STI diagnosed during the previous half year(n = 768)					
No	631(82.2)		137(21.7)	1	
Yes	137(17.8)		48(35.0)	1.95(1.31–2.90)	0.000
STI risk perception					
Impossible/unsure	1785(67.5)		299(16.8)	1	
Possible	860(32.5)		280(32.6)	2.40(1.99–2.90)	0.000
HIV risk perception					
Impossible/unsure	2052(77.6)		394(19.2)	1	
Possible	591(22.3)		185(31.3)	1.92(1.56–2.36)	0.000
Scale for self-efficacy for condom use					
0	1027(38.8)		280(27.3)	1	
1–2	358(13.5)		73(20.4)	0.68(0.51–0.92)	0.010
3	1260(47.6)		226(17.9)	0.58(0.48–0.71)	0.000

*OR = odd ratio; CI = confidence interval.

Those who engaged in commercial sex in two counties (OR = 2.08, 95% CI = 1.60–2.08), those working in three or more counties (OR = 3.94, 95%CI = 3.15–4.94) versus those engaging in commercial sex in one county; those who had engaged in commercial sex for 13–24 months (OR = 3.22, 95% CI = 2.45–4.24), and for over 24 months (OR = 2.71, 95% CI = 2.17–3.39) versus those who had worked for 1–12 months; those who had experienced commercial sex with 16–30 partners (OR = 4.06, 95% CI = 3.17–5.21), and over 30 partners (OR = 2.68, 95% CI = 2.06–3.47) versus those who had had fewer partners; those who had experienced anal sex versus those who had not (OR = 11.35, 95% CI = 7.64–16.85); those who had shown STI-related symptoms during the previous 6 months (OR = 2.11, 95% CI = 1.64–2.71) versus those who had not; those diagnosed with an STI versus those not (OR = 1.95, 95% CI = 1.31–2.90) among those who had seen a doctor for STI diagnosis and treatment during the previous 6 months; those who believed that they were likely to contract STIs versus those who believed it was unlikely or unsure that they would contract STIs (OR = 2.40, 95% CI = 1.99–2.90); those who believed that it was likely that they might contract HIV versus those who believed it was impossible or who were unsure whether they might contract HIV (OR = 1.92, 95% CI = 1.56–2.36), were more likely to engage in oral sex. Having seen a doctor during the previous six months was not associated with experience of oral sex.

### Multivariate analysis

Controlling for possible confounding variables, multivariate analysis revealed that those FSWs who were married or cohabiting (OR = 0.65, 95%CI = 0.48–0.89), and those who were widowed or divorced (OR = 0.56, 95%CI = 0.34–0.92) versus those who were unmarried, earned an income of 3000–4000 RMB (OR = 0.59, 95% CI = 0.44–0.78), and over 4000 RMB (OR = 0.63, 95% CI = 0.47–0.85), those who had initiated sex at the age of 26–30 years (OR = 0.62, 95%CI = 0.43–0.89) or over 30 years (OR = 0.37, 95% CI = 0.24–0.56) versus those who had initiated sex at age 20 or less; those who always used a condom versus those who never/sometimes used a condom (OR = 0.55, 95% CI = 0.41–0.75); those who always used oral contraceptive versus those who never/sometimes used it (OR = 0.39, 95%CI = 0.22–0.67); and those who used contraception versus those who did not (OR = 0.70, 95% CI = 0.54–0.90), continue to be less likely to use condoms (Table 4).

**Table 4:** Multivariate analysis for association of oral sex.

Variable	Adjusted OR (95%CI) *	P value
Marital status		
Unmarried	1	
Married/cohabited	0.65(0.48–0.89)	0.006
Widowed/divorced	0.56(0.34–0.92)	0.021
Income of a family		
<3000	1	
3000–4000	0.59(0.44–0.78)	0.000
>4000	0.63(0.47–0.85)	0.003
Age of first commercial sex		
<21	1	
21–25	0.76(0.57–1.02)	0.064
26–30	0.62(0.43–0.89)	0.009
>30	0.37(0.24–0.56)	0.000
County number for commercial sex		
1	1	
2	1.59(1.17–2.16)	0.003
>2	2.13(1.60–2.84)	0.000
Duration of commercial sex		
1–12 months	1	
13–24 months	2.47(1.78–3.42)	0.000
>24 months	2.32(1.73–3.11)	0.000
Number of commercial client during the previous one month		
<16	1	
16–30	2.95(2.22–3.93)	0.000
>30	2.19(1.60–3.00)	0.000
Anal sex during the previous one month		
No	1	
Yes	9.42(6.00–14.80)	0.000
Condom use during the previous one month		
Never/sometimes	1	
Always	0.55(0.41–0.75)	0.000
Oral contraceptive use during the previous one month		
Never/sometimes	1	
Always	0.39(0.22–0.67)	0.001
Contraception measures at present time		
No	1	
Yes	0.70(0.54–0.90)	0.006
STI related symptom during the previous half year		
No	1	
Yes	1.57(1.15–2.13)	0.004

*OR = odd ratio; CI = confidence interval.

Those FSWs who engaged in commercial sex in two counties (OR = 1.59, 95% CI = 1.17–2.16) and those working in three or more counties (OR = 2.13, 95%CI = 1.60–2.84) versus those engaging in commercial sex in just one county; those who had engaged in commercial sex for 13–24 months (OR = 2.47, 95% CI = 1.78–3.42), or for over 24 months (OR = 2.32, 95% CI = 1.73–3.11) versus those who had worked for 1–12 months; those who had experienced commercial sex with 16–30 partners (OR = 2.95, 95% CI = 2.22–3.93), and with over 30 partners (OR = 2.19, 95% CI = 1.60–3.00) versus those who had had fewer partners; those who had experienced anal sex versus those who had not (OR = 9.42, 95%CI = 6.00–14.80); and those who had shown STI-related symptoms during the previous 6 months (OR = 1.57, 95%CI = 1.15–2.13) versus those who had not, were more likely to engage in oral sex.

## Discussion

To the best of our knowledge, this study is the first research of its kind to examine factors associated with oral sex among low-tier FSWs in China. We reported that the prevalence of oral sex practiced by low-tier FSWs in this study was 21.9%. Since we only measured this prevalence during the previous month, we are not able to compare this result with overseas FSWs [14, 15] or those in China [16], nor could we compare these data with those of common Chinese people [18, 19], since previous studies have all used measures based on lifetime prevalence. However, we believe that the relatively large sample size and relatively high prevalence of oral sex during the previous month among our participants could guarantee the generalizability of our study findings, and the comparability between the results of this study and other related literature. Another reason for the incomparability of the prevalence of oral sex between this study and other related studies is that these studies were conducted in different years following a revolution in sexual practices in China since 2000, and the subsequent dramatic increase in sexual behaviors traditionally adopted by few common people in China, such as oral and anal sex [19]. We speculate that the prevalence of oral sex among FSWs might also have increased quickly due to this trend in these years. The amazing rise in prevalence of oral sex among FSWs, with a concomitant increase in pharyngeal gonorrhea, has been reported in Singapore [28], pointing to the importance of this study.

Our study found that 2.2% of oral sex practitioners only practiced oral sex with their clients; the remaining majority have not only engaged in oral sex, but also in vaginal and/or anal sex, indicating that oral sex is just one type of commercial sexual service provided to clients. Previous studies have shown that low-tier FSWs are at a lower socio-economic level than higher-tier FSWs [5–7]. The income of those low-tier FSWs who practice oral sex is lower than those not performing oral sex in our study, implying that the economic situation of oral sex practitioners might be worse than that of other low-tier FSWs. This suggests that, due to economic reasons, they might be more willing to provide oral sex, or unable to reject clients’ requests for oral sex, anal sex, and unprotected sex.

Low-tier FSWs were more mobile than other economic levels of FSW [29]. We found that approximately 56% of low-tier FSWs engaged in commercial sex in two or more counties, and the more likely they were to perform oral sex for clients, the more counties in which they perform commercial sex. FSWs were highly mobile, with reasons for changing residence and working locations most commonly related to increasing income. FSWs working in higher risk venues where they charge less have many characteristics in common with HIV positive and drug using FSWs, who have been found to be more mobile than those from other establishments [11]. Mobility among FSWs has been assumed to contribute to the spread of HIV, and HIV may spread to low-risk areas through mobile FSWs [30, 31]. Thus, importance should be attached to the mobility of FSWs who practice oral sex, particularly to reduce their risk of HIV/STI transmission in behavioral intervention, and to provide information about support facilities such as VCT clinics in different areas.

Our results indicate a significant association between occurrence of oral sex and longer duration of sex work. Prolonged length of sex work might increase the likelihood of oral sex; however, longer duration of sex work is an independent risk factor for STIs [32] and HIV infection [33]. Though some foreign studies have conversely documented that STI infections are higher in those with a shorter compared to those with a longer period of sex work [34–36], the risks of HIV/STI infection resulting from long duration of sex work among FSWs who practice oral sex should be noted in the Chinese context. We further compare the difference between those FSWs who engaged in commercial sex for 1–12 months and those who have practiced for over 12 months, and found that FSWs who had been engaged in sex work for a long duration were more likely to have had over 15 sexual clients (72.3% Vs. 48.3%) and never/sometimes use a condom (53.9% Vs. 41.3%) during the previous month, to report STI-related symptoms (13.4% Vs.9.9%), and to have been diagnosed with an STI in those who had seen a doctor (21.8% Vs. 12.3%) during the previous half year.

We found that those who practiced oral sex were more likely to report ever having been diagnosed with an STI among FSWs who had seen a doctor during the previous half year in the bivariate analysis. This study provides further evidence that oral sex practitioners are more likely to report STI-related symptoms. Low-tier FSWs are more likely to report STIs [24–26] and genital symptoms [29] than higher-tier FSWs. We concluded, therefore, that of low-tier FSWs, oral sex practitioners are more vulnerable to STI infection than those who do not practice oral sex.

It is well understood that the risk of HIV infection and transmission during anal sex is significantly higher than during vaginal sex [37–39]. Though only 5% of our study participants reported practicing anal sex with their clients during the previous month, anal sex practice is the strongest predictor of oral sex in this study. These FSWs might conduct both oral and anal sex with their clients because they are more obedient to client requests in order to secure a deal in the sex trade, emphasizing that HIV prevention interventions should not ignore those practicing oral and anal intercourse to prevent HIV transmission.

Our study demonstrated that oral sex is a strong indicator of lower age of first instance of commercial sex, consistent with a previous Chinese study that oral sex practitioners are more likely to initiate sex at a younger age [17]. Sex initiation at a lower age has been confirmed to be related to having more sexual partners, having sex more frequently, diagnosis with an STI, history of pregnancy, induced abortion, and less condom and oral contraceptive use, compared with late initiators [40, 41]. Early age of sex initiation is also a predictor of HIV infection among sex workers in the Chinese literature [42]. These early initiators of commercial sex might be at greater risk of HIV/STI transmission, unwanted pregnancy, and induced abortion than other FSWs, and this should be considered an important indicator for intervention in low-tier FSWs.

There is more evidence to support the above arguments. In this study, oral sex practitioners are related to less use of condoms or oral contraceptives during the previous month and contraception use. We reported that only approximately 8% of oral sex practitioners always use oral contraceptives, 16% had always used condoms during the previous month, and 20% were currently adopting contraception measures. We reported that approximately 80% of oral sex practitioners are aged under 35 years, and they are more likely to be unmarried. Oral sex practitioners are mostly at the reproductive age. These results indicate a relatively low awareness of reproductive health, and a large unmet need in this respect in this group of low-tier FSWs, underscoring the need to expand FSW-friendly reproductive health services in Zhejiang province. The structural interventions, such as increasing condom availability in sex trade establishments, distribution of condoms free of charge by outreach workers, accessible reproductive-health services, and provision of reproductive health education may improve their reproductive health outcomes.

Sixteen percent of oral sex practitioners had used condoms consistently during their commercial sex in the previous month versus 51.2% of the total participants, and oral sex is significantly related to less condom use in this study. However, our multivariate analysis indicates that oral sex practitioners did not increase their likelihood of self-efficacy for condom use and risk perception for HIV and STI infection. Low condom use is common among FSWs in China [16]. The main purpose for which FSWs use condoms is for contraception, not for prevention of HIV and other STIs [43]. Only a small proportion of FSWs have access to condoms at their sex trade places [44], with the majority of women having to buy condoms themselves [16]. Half of FSWs had had the experience of condom breakage or slippage during sex [16]. Their lack of negotiating power and fear of losing clients resulted in low condom use among FSWs [45]. Low-tier FSWs reported a lower rate of condom use than higher tier FSWs, perhaps driven by the desire for more clients and higher trade fees, and hampered by their limited ability to negotiate for safer sex practices [8, 9, 29, 46]. Therefore, comprehensive intervention with multiple components, including addressing the barriers to condom use mentioned above, should be carried out among oral sex practitioners of low-tier FSWs. Multiple session interventions may be required as the condom use is so low in this group, and multiple session intervention has been found to be more effective than single session intervention for increasing condom use among FSWs [47].

We are not clear about the rate of condom use in oral sex, but could speculate that oral sex is much less protected than other sex practices. Foreign studies have shown that oral sex among FSWs and their clients is less protected [14, 15]. One Chinese study revealed that 70.1% of FSWs always used condoms to protect themselves when they had vaginal sex with clients, and this figure was 57.9% for anal sex but only 22.7% for oral sex [16]. Those oral sex practitioners are at risk of HIV/STI infection through oral sex, vaginal sex, and anal sex. Oral sex is also associated with more commercial clients in this study. These FSWs also place more clients at risk of infection once infected.

This study offers new insights into the oral sex related factors among Chinese FSWs. Our study is subject to several limitations, however. First, the cross-sectional nature of this study did not allow cause-and-effect relationships to be established between low-tier FSWs engaged in oral sex and risk behaviors. Second, we conducted this investigation in 21 counties during a limited time period, and the FSWs included in this study may have been more accessible and more willing to participate. The results presented in this article may not be generalizable to the whole province or to China. Third, our findings may be limited by the validity of self-reported measures, as our questionnaire contained some sensitive sexual behavior questions [48], and social desirability may have resulted in over- or under-reporting of sexual behaviors. Fourth, recall bias may occur to FSWs in our study, due to their education status and working environment, though we tried to interview them on sexual behaviors mainly in the previous month.

## Conclusion

In conclusion, our study reported that low-tier FSWs who engaged in oral sex exhibited a profile of higher-risk behaviors such as early initiation of commercial sex, being more mobile, longer duration of sex work, more commercial clients, lower use of condoms and contraceptives, no use of contraception, and a reported history of STI symptoms compared with other low-tier FSWs. They not only place themselves, but also their commercial clients and regular partners at greater risk of STI/HIV infection and reproductive health problems. Prevention programs targeting low-tier FSWs working on streets, at hair salons, in roadside shops, and in other venues should specifically target low-tier FSWs who practice oral sex, should carefully take into account the characteristics of these FSWs, provide risk awareness education and training for condom use negotiation, and promote the availability of condom and reproductive health care, timely diagnosis, and treatment of STIs.
